# Validation of Reference Genes for Robust qRT-PCR Gene Expression Analysis in the Rice Blast Fungus *Magnaporthe oryzae*

**DOI:** 10.1371/journal.pone.0160637

**Published:** 2016-08-25

**Authors:** Sarena Che Omar, Michael A. Bentley, Giulia Morieri, Gail M. Preston, Sarah J. Gurr

**Affiliations:** 1 Department of Plant Sciences, University of Oxford, South Parks Road, Oxford, OX1 3RB, United Kingdom; 2 BioSciences, Geoffrey Pope Building, University of Exeter, Exeter, EX4 4QD, United Kingdom; University of Nebraska-Lincoln, UNITED STATES

## Abstract

The rice blast fungus causes significant annual harvest losses. It also serves as a genetically-tractable model to study fungal ingress. Whilst pathogenicity determinants have been unmasked and changes in global gene expression described, we know little about *Magnaporthe oryzae* cell wall remodelling. Our interests, in wall remodelling genes expressed during infection, vegetative growth and under exogenous wall stress, demand robust choice of reference genes for quantitative Real Time-PCR (qRT-PCR) data normalisation. We describe the expression stability of nine candidate reference genes profiled by qRT-PCR with cDNAs derived during asexual germling development, from sexual stage perithecia and from vegetative mycelium grown under various exogenous stressors. Our Minimum Information for Publication of qRT-PCR Experiments (MIQE) compliant analysis reveals a set of robust reference genes used to track changes in the expression of the cell wall remodelling gene *MGG_Crh2* (MGG_00592). We ranked nine candidate reference genes by their expression stability (M) and report the best gene combination needed for reliable gene expression normalisation, when assayed in three tissue groups (Infective, Vegetative, and Global) frequently used in *M*. *oryzae* expression studies. We found that *MGG_Actin* (MGG_03982) and the 40S 27a ribosomal subunit *MGG_40s* (MGG_02872) proved to be robust reference genes for the Infection group and *MGG_40s* and *MGG_Ef1* (Elongation Factor1-α) for both Vegetative and Global groups. Using the above validated reference genes, *M*. *oryzae MGG_Crh2* expression was found to be significantly (p<0.05) elevated three-fold during vegetative growth as compared with dormant spores and two fold higher under cell wall stress (Congo Red) compared to growth under optimal conditions. We recommend the combinatorial use of two reference genes, belonging to the cytoskeleton and ribosomal synthesis functional groups, *MGG_Actin*, *MGG_40s*, *MGG_S8* (Ribosomal subunit 40S S8) or *MGG_Ef1*, which demonstrated low M values across heterogeneous tissues. By contrast, metabolic pathway genes *MGG_Fad* (FAD binding domain-containing protein) and *MGG_Gapdh* (Glyceraldehyde-3-phosphate dehydrogenase) performed poorly, due to their lack of expression stability across samples.

## Introduction

Rice blast disease is caused by the ascomycete fungus *Magnaporthe oryzae* (previously known as *Magnaporthe grisea* [1]). Despite active pathogen management strategies, such as the deployment of disease resistant cultivars and the widespread spraying of antifungals, year-on-year crop losses occur. Indeed, recent rice blast outbreaks have been seen in USA, Thailand, China, India and Japan [2–5]. With significant annual yield losses (up to 30%) attributable to this fungus alone, and as the global demand for rice increases alongside population growth, better control of this disease is imperative [6–8]. Recent years have seen growing interest in the fungal cell wall as a potential phylum-specific target for disease mitigation. In this regard, our quest is to describe the mechanisms which underpin cell wall construction and which endow it with sufficient plasticity to support expansion, growth and infection [9–11]. To achieve such plasticity, specialised groups of GPI-anchored glycosyltransferase (GST) proteins such as Gasp/Gelp, [12–14], Crh1p/Crh2p [15, 16], Dfg1p/Dcw5p [17] and Sps2p (Ecm33p) [18] continuously remodel synthesised polysaccharides in response to changes in the external environment or with the developmental clock [12, 13, 16, 17]. Of the various GSTs, the *Crh*-like family of Crhp, carrying Crh1p, Utr2p and Crr1p was first described in *Saccharomyces cerevisiae* [19]. These proteins share a functional domain belonging to the Glycosyl Hydrolase 16 group of Carbohydrate Active Enzymes (CaZy) and catalyse the hydrolysis of β-(1,4) linkages in the chitin polymer and the transfer of chitin to a glucan acceptor, usually to either β-(1,3) or (1,6) glucan chains, and subsequent branch formation with chitin polymers [20, 21]. The cross-linking of these alkaline-insoluble chitin chains to the soluble glucan polymers is responsible for providing cell wall rigidity and strength [22, 23] and therefore inactivation of the Crh-like proteins could potentially influence fungal pathogenicity. In agreement with this, *Candida albicans* knockout mutant strain ΔUtr2 was less virulent and unable to colonize mice organs [24]. Furthermore, Pardini et al. [15] then developed a triple mutant which was fully non-pathogenic even when mice were infected at a lethal dose. Results from the studies above are an important indication that targeting Crh-like genes has the potential to impair a pathogen’s viability, subsequently affecting its ability to infect and/or colonize a host.

Little is known about this family of genes in plant pathogenic fungi. As a first step, we set out to capture the expression profile of *M*. *oryzae MGG_Crh2* (orthologous to *Crh1* in *S*. *cerevisiae*) during fungal development (asexual morphogenesis, sexual structure formation and vegetative growth) and when grown under cell wall stress (Congo Red). This undertaking posed several technical challenges, not least the paucity of pathogen RNA present within the host tissues (often <0.1%). In *S*. *cerevisiae*, temporal expression of *Crh1* increases by 2-fold as the yeast enters the budding phase, whilst under cell wall stress, invoked by Congo Red, Calcofluor White or Zymolyase, expression increases by 2–4 fold [25, 26]. Despite these small fold changes, yeast *Crh* mutants exhibit hypersensitivity to these cell wall perturbing compounds, as well as altered cell surface plasticity [22]. Such small fold changes in protein transcript abundance further re-enforce the need for a sensitive, reliable and robust quantification technique.

A multitude of publications attest to the usefulness of both individual and genome-wide gene expression studies in *M*. *oryzae*. However, whilst large-scale transcript analyses with microarrays and/or high throughput-SUPERSAGE have captured gross fold-changes over several thousand genes [27, 28], such analyses are not sensitive enough to detect small changes in low abundance genes. Moreover, they are overly challenging or, indeed, impossible to perform with minute samples of tissue-specific RNA. Quantitative Real-Time PCR provides a low throughput (*ie* a restricted number of genes) but highly sensitive technique [29, 30]. Indeed, it has been shown to be 5-fold more sensitive than microarray data [31]. However, to obtain reliable data, qRT-PCR requires stringent preparation strategies, including good quality RNA samples and the use of efficient and specific primers [32–34]. In addition, the choice and number of reference genes used for data normalisation are critical for robust analysis of gene expression [34, 35]. Studies have shown that expression stability of a reference gene varies between species, and that expression could also vary across sample tissue type and experimental conditions [32, 33, 36, 37]. Moreover, using an inappropriate number of reference genes can adversely affect data reliability–as shown with *Drosophila melanogaster* head cDNA, where the relative expression of genes *GSTD1*, *INR* and *HSP70* differed significantly when the normalising factor (NF) used either 1, 3 or 13 reference genes [38]. As such, the use of un-validated and/or single reference genes in qRT-PCR assays, such as, for example, *MGG_β-Tubulin*, *MGG_Actin*, *MGG_Gapdh* or *MGG_Ubiq* in *M*. *oryzae* is strongly discouraged. In addition, sample quality control, validation, and assay optimisation prior to running the qRT-PCR in gene expression assays are important for data reliability. Recognising this, recently there has been an increase in the number of reference gene validation studies in plant fungal pathogens such as in *Puccinia* sp. (wheat rust) [39], *Fusarium* sp. (wheat head blight) [40], and *Aspergillus* sp.(Black mould) [41]. However, prior to the work conducted by Park et al. [27], studies of gene expression in *M*. *oryzae* used only one, un-validated reference gene such as *MGG_β-Tubulin* [42, 43], *MGG_Actin*, or *MGG_Gapdh* [44]. To redress this, Park et al. [27] recently attempted to validate seven candidate genes, identifying *MGG_β-Tubulin* as the most stably-expressed gene of their cohort. The work, however, comes short of identifying the appropriate number of reference genes to use.

Through the emphasis for stringent qRT-PCR preparation, this paper was prepared in accordance to the guidelines made for the Minimum Information for Publication of qRT-PCR Experiments (MIQE) [34]. Furthermore, given the need to improve current measurement of gene expression practices in *M*. *oryzae* studies, this paper utilises additional candidate reference genes as well as genes commonly used in this field of study. Here, nine candidate reference genes from various functional groups were shortlisted, the stability validated and importantly, the appropriate number of genes needed for normalisation of gene expression under various fungal growth conditions were determined in this study. We then used the chosen reference genes to investigate the expression profile of *MGG_Crh2*, a cell wall remodelling protein, during various developmental stages and growth under various exogenous stresses. Thus, we have evaluated and determined the optimal number of reference genes to use, but have also appraised and added several further candidate genes, from disparate functional groups that out-perform the expression stability of *MGG_β-Tubulin* as the ideal reference gene. We hereby propose a robust qRT-PCR data normalisation strategy for transcript analysis in *M*. *oryzae* that will improve the confidence and reliability of all future gene expression analysis work related to this fungus.

## Materials and Methods

### Fungal Strains and Growth Conditions

Rice blast fungus *Magnaporthe oryzae* (*M*. *grisea* (T.T. Herbert) M.E. Barr) wild type (WT) strains Guy11 (MAT1-2) and TH3 (MAT1-1) were cultured on complete medium (CM) at 24°C, 14 hour light 10 hour dark cycle. Strain maintenance and media composition were as described by Talbot et al. [45]. Perithecia were produced by crossing Guy 11 with the opposite mating type strain TH3: agar plug inocula of the 2 strains were placed 4 cm apart on oatmeal agar and incubated under constant fluorescent light at 18°C for 29 days. Perithecia were then harvested using a dissecting microscope and snap-frozen in liquid nitrogen. Guy11 spores were harvested from 10-day-old CM plates by scraping the surface with 10 mL deionised water with a glass slide, and poured through triple layered Miracloth (Merck Chemicals Ltd, Padge Rd, Nottingham, NG9 2JR, UK). Spore concentrations were adjusted using a haemocytometer prior to use.

### Tissue Preparation for RNA Extraction

#### Mycelial tissue grown under various stress factors

Two hundred μL of 4 x 10^5^ spores/mL Guy11 spore suspension was added to 500 mL of various liquid cultures, and incubated at 24°C for 3 days in the dark, under continuous shaking (150 rpm). Mycelia were harvested by filtering through double-layered Mira-cloth, dried with paper towels and 0.1 g of fungal mycelia was snap frozen in liquid nitrogen and stored at -80°C. The process was repeated to gather three biological replicate samples for each condition assayed.

For control vegetative growth under optimal conditions, *M*. *oryzae* Guy11 was cultured in Complete Medium (CM). Starvation was achieved by culturing in Minimal Medium (MM) made from 1x nitrate salts, 1% D-glucose, 0.1% ml trace elements solution, 0.1% thiamine solution, and 0.05% biotin solution. In extreme starvation, glucose was omitted from the Minimal Medium preparation above (MM-glucose). For cell wall stress, the cell wall perturbant Congo Red (SIGMA-Aldrich) was prepared in a 1% stock solution, filter-sterilised and added to sterile Complete Medium buffered with 50 mM HEPES (pH 7.0) at a concentration of 100 μg/mL. Stress by caffeine was induced by adding 2.5 mM filter-sterilised caffeine (SIGMA-Aldrich) to sterile Complete Medium (pH 6.5). To impose osmotic stress, 1 M Sorbitol was added to Complete Medium (pH 6.5) prior to autoclaving.

#### Dormant spores at 0 hours post inoculation (hpi)

Spores were harvested from 10-day-old WT strain Guy11 grown on Complete Medium agar plates, 25 plates per biological replicate (three biological replicates harvested separately). The combined spores were centrifuged (5 minutes at 13, 000 rpm, 4°C), the supernatant removed and the pellet snap frozen in liquid nitrogen.

#### Germling development at 2, 8, 24, and 48 hours post inoculation (hpi) on host

Barley seeds (*Hordeum vulgare* L.) cv. Golden Promise were grown in a 50:50 (w/w) mixture of Erin Multipurpose Compost and John Innes No. 2 soil-based compost, for 7 days at 25°C, 12/12 light, 70% humidity. Seven-day-old barley leaves were cut and placed onto 1.5% (w/v) water agar and inoculated with harvested spores (6–7 x 10^6^ spores/mL) re-suspended in 0.4% (w/v) gelatine. Inoculation procedure: for each time point, 2.5 mL of the spore suspension was sprayed uniformly onto 24 leaves, which were then covered to maintain high humidity and incubated at 25°C for 2, 8, 24 or 48 hours post inoculation. The hours post inoculation (hpi) corresponds to the various infective stages: 2 hpi (germ tube emergence), 8 hpi (immature appressoria), 24 hpi (penetration and early invasive growth), and 48 hpi (*in planta* growth). Prior to harvest, one leaf was selected at random, and its surface viewed by light microscopy to monitor germling development and formation of appressoria. Approximately 0.1 g of leaf material was then snap frozen in liquid nitrogen and stored at -80°C, for RNA extraction. The process was repeated, harvesting three biological replicates.

#### Perithecia

Guy11 and TH3 were inoculated onto oatmeal (10% (w/v)) agar and incubated as described previously. Perithecia (0.1 g) were harvested, snap frozen in liquid nitrogen and kept stored at -80°C prior to RNA extraction. Three biological replicates were harvested independently.

### RNA Extraction, Quantity, and Quality Test

Tissues were extracted with TRIzol® Reagent (Ambion®) using the manufacturers’ instructions, followed by the addition of 50% volume isopropanol, with 5 minutes incubation at room temperature. Samples were loaded into an RNA Quick Spin column (Qiagen RNeasy Minikit) for on-column DNAse digestion and subsequent elution in RNase-free water. RNA sample concentration was determined using the Thermo Scientific ‘NanoDrop® ND-1000’ spectrophotometer. Samples were normalized to the same concentration of 1000 ng/μL (±100 ng). RNA quality and RIN number for each sample were tested using the ‘Agilent 2100 Bioanalyzer Instrument’ as per the manufacturers’ instructions to ensure minimal RNA degradation. Sample RNA was discarded if the RIN value was below 7.5 for samples containing only fungal RNA, and if the RIN value was below 6.0 for samples containing a mix of plant leaf and fungal pathogen RNA.

### cDNA Preparation

Qiagen ‘Maxima First Strand cDNA Synthesis Kit for RT-qPCR’ kit was used as per manufacturers’ instructions. The same amount of template RNA (1.0 μg), was added per 20 μl reactions for each tissue sample. Three controls were prepared: Non-template control (NTC), minus Reverse Transcriptase (-RT) and RNA from Barley leaf only (BL). The reaction mix was incubated for 10 min at 25°C, followed by 30 min at 50°C, and the reaction terminated by heating at 85°C for 5 min. Products were retained at -20°C and used within 2 weeks.

### Candidate Reference Gene Selection and Primer Design

Candidate reference genes were selected on the basis of being (a) previously used in *M*. *oryzae* gene expression studies [42, 43, 46–48], and (b) from several essential functional groups (protein translation, glycolysis pathway, cytoskeleton). These genes were then cross-checked with the publicly available COGEME high throughput-super SAGE transcript profile *M*. *oryzae* database [49], for an estimation of stability. Stability ratios were calculated using the given base mean values between samples of the same growth stage (example: Complete Medium against Minimal Medium, or 4 hpi against 16 hpi). Genes with a ratio less than 10 from the transcriptomic database were then selected for further analysis. Sequences for the respective genes were obtained from the 8^th^ annotation (MG8) *Magnaporthe oryzae* strain 70–15 sequenced by Broad Institute [30]. All primer pairs (Table 1) were synthesized by SIGMA-Aldrich and designed according to the following specifications: annealing Tm 60°C (±2°C); G-C content 50–60%; no non-specific product amplification; no primer-dimer or secondary structures; amplicon of 60–110 bp; primer efficiency 80–110%, r^2^ value 0.98–0.99; either Forward or Reverse primer to span exon/exon boundary (transcript-specific). Primer specificity: primers were deemed acceptable if the targeted gene showed an e value less of than 10^−3^ in NCBI Blast search. In addition, primer pairs were shown to be pathogen-specific by conducting qRT-PCR using only host barley leaf cDNA as a negative control (BL). Primers were tested for non-specific product/s by amplicon separation on 2% (w/v) agarose gel electrophoresis and 10–300 bp GeneRuler Ultra Low Range DNA Ladder (Thermo Scientific) at the end of a qRT-PCR run. [50]

**Table 1:** Information regarding genes used and primers designed for this study.

Gene name	Gene ID and accession num	Symbol used	Forward Primer*	Reverse Primer*	Size (bp)	Efficiency (E)	R^2^ Value	Reference
β-Tubulin chain	MGG_00604 (XM_003718381)	*MGG_β-Tubulin*	CTGCCATCTTCCGTGGAAAGG	GACGAAGTACGACGAGTTCTTG	86	1.95	0.9998	[50]
Elongation factor 1-α	MGG_03641 (XM_003716200)	*MGG_Ef1*	CATCTTAACGTCGTCGTCATC	AGTGGCCGGTAGTCGTGG	62	1.92	0.9995	This study
Actin	MGG_03982 (XM_003719823)	*MGG_Actin*	ACAATGGTTCGGGTATGTGC	CGACAATGGACGGGAAGAC	76	2.02	0.9975	This study
Ubiquitin-conjugating enzyme	MGG_04081 (XM_003719709)	*MGG_Ubiq*	ATCCTAATGTCTACCCGAG	GATGCGTGTTCGTAGTGG	85	1.99	0.9996	This study
Glyceraldehyde-3-phosphate dehydrogenase	MGG_01084 (XM_003717805)	*MGG_Gapdh*	CAAGTACGCCAAATACATGC	TTGCCGTTGACGACCAGG	96	2.06	0.9988	This study
40S ribosomal protein subunit S27a	MGG_02872 (XM_003720814)	*MGG_40S*	ACAAGCTCAAGACCCTCGTC	GGTGGTGATGGTGAAGCAG	80	1.95	0.9996	This study
40S ribosomal protein subunit S8	MGG_03251 (XM_003716675)	*MGG_S8*	GCTCACTACCGCCAGAAGC	ACGGACGGTGTGAATGCG	87	1.90	0.9989	This study
FAD binding domain-containing protein	MGG_01605 (XM_003714541)	*MGG_Fad*	ACCTTGTTGGCTGCGATG	TCCATCGAGTACACCCCAC	104	2.04	0.9984	This study
Nucleolar Protein	MGG_07008 (XM_003709718)	*MGG_Nuc*	AGTGCGGGTTATGGTCTCTTC	CTGCTCTCCAGGTCATCTGC	64	2.04	0.9978	This study
Crh2 cell wall *glycosyltransferase* gene	MGG_00592 (XM_003718400)	*MGG_Crh2*	GAGATCGACTGGGAGCACG	AGACGGTGTCATTACCCTTGG	79	1.98	0.9955	This study

*underlined nucleotides represent the second exon in a primer that anneals to an exon/exon boundary

### Quantitative Real Time PCR (qRT-PCR)

Real time PCR for the Infective group was conducted using an Mx3000P qPCR System (Agilent Technologies), which was replaced with an ABI 7000 apparatus (Applied Biosystems) for the Vegetative and Global group. All reactions were conducted in 96-well plates (MicroAmp® Optical 96-Well Reaction Plate cat N8010560) utilising a sample maximisation arrangement and the addition of inter-run calibration (IRC) wells for each gene in each 96-well plate [51]. ‘Power SYBR Green PCR Master Mix’ assay (Applied Biosystems) was used in a total volume of 25 μL per well/reaction. Each 25 μL reaction contained 2.0 μL cDNA, 8.5 μL water, 1.0 μL (10 μM) forward primer, 1.0 μL (10 μM) reverse primer and 12.5 μL ‘Power SYBR Green PCR Master Mix’. All qRT-PCR plate amplifications followed the thermal cycler steps: 50°C for 2 minutes followed by 95°C for 10 minutes; 95°C 15 seconds, followed by 60°C annealing temperature for 1 minute, repeating this final step for 40 cycles. Fluorescence of the ‘SYBR Green I’ dye was calibrated against ROX™. At the end of each reaction, a dissociation curve analysis was performed, with the following thermal profile: 95°C for 15 seconds; 60°C for 30 seconds followed by 95°C for 15 seconds. Data from each group of tissue type (Infective, Vegetative, and Global) was analysed independently (both geNorm and expression profile).

### Primer Efficiency, Specificity, and Genomic DNA Contamination Check

Complementary DNA (cDNA) from all samples was pooled, diluted 5-fold and used as templates. Cycle threshold (Ct) values were analysed as follows: the primer efficiency (E) was calculated from the four point slope of the plotted dilution row, using the formula E = 5^^(1/S)^ whereby S is the slope of the regression line. Primer pairs with slope r^2^<0.99, E <1.8 or dissociation curve and gel electrophoresis runs showing non-specific product/s, were rejected and re-designed, prior to gene stability analysis. Primer efficiency data is shown in Table 1. For further quality control purposes, samples with technical replicates showing more than 0.8 difference in Ct value, or occurrence of amplification in non-template and host-only control (BL) with Ct values less than 35, were rejected.

### Evaluation of Reference Gene Expression Stability Using GeNorm Analysis

Analysis of raw Ct data for all candidate reference genes was performed using ‘qBase+ Basic’ licensed software (Biogazelle). Each group (Infective, Vegetative, and Global) was analysed separately. The Infective group included tissues from dormant spores (0 hpi) and at various time points corresponding to different *M*. *oryzae* infective stages: germ tube (2 hpi), immature appressoria (8 hpi), mature appressoria (12 hpi), penetration peg and haustoria (24 hpi), and extensive *in planta* growth (48 hpi). The Vegetative group included vegetative stage fungal mycelia harvested from an optimal growth condition (CM) and under various environmental stresses such as starvation (MM), extreme starvation (MM-glucose), osmotic stress (Sorbitol), cell wall stress (Congo Red) and caffeine stress. The Global group included a mixture of the above: dormant spores, immature appressoria (8 hpi), CM, starvation (MM) and perithecia (sexual reproductive stage).

For each group (Infective, Vegetative or Global), raw ct values were converted to relative expression values using the Pfaffl Method [52] and inter-run calibration was performed to correct for run-to-run variation [51]. The GeNorm algorithm [53] in ‘qBase+ Basic’ licence software version 2.5 (Biogazelle) was used to calculate the average expression stability score (M) for each reference gene, and the pairwise variability score (V) for sequential normalisation factors, resulting from stepwise inclusion of additional reference genes. Expression stability values were used to identify the most stably-expressed reference genes at each stage, whilst pairwise variability scores were used to determine the most appropriate number of reference genes to use as the normalisation factor.

### Gene of Interest (GOI): Orthologue of *S*. *cerevisiae Crh1* gene

The *Crh1* gene was first identified in the model yeast *S*. *cerevisiae* [19]. The protein sequence was obtained from the Saccharomyces Genome Database [54] and the corresponding orthologue in *M*. *oryzae* was obtained from “Magnaporthe comparative Sequencing Project” by the Broad Institute of Harvard and MIT [30] using the BLASTp search tool [55]. Here, the yeast Crh1 protein sequence was queried against proteins predicted to be encoded by the *M*. *oryzae* 70–15 (MG8) sequenced genome. The best hit genes with a cut-off value of e^-10^, were then reciprocal BLASTp [55] against *S*. *cerevisiae* genome in the NCBI database (www.ncbi.nlm.nih.gov).

### Data analysis: Expression Profile of *MGG_Crh2* Across the *M. oryzae* life-cycle

The validated combination of reference genes according to tissue types were used as Normalising Factor (NF) to analyse the expression profile of a cell wall remodelling enzyme, *MGG_Crh2* (MGG_00592) under various stages of the life cycle (Infective and Global assay) and growth conditions (Vegetative assay). In the latter, the expression of *MGG_Crh2* under starvation (MM), osmotic stress (Sorbitol), extreme starvation (MM-glucose), and cell wall stress imposed by Congo Red (CR) or caffeine was measured relative to vegetative growth in nutrient-rich medium (CM). In the Infective assay, expression profile was measured during germling development for tissues harvested from 2 hpi (germ tube), 8 hpi (immature appressoria), 24 hpi (penetration and early invasive growth) and 48 hpi (*in planta* growth), relative to 0 hpi (dormant spores). Finally, the expression profile of *MGG_Crh2* was captured across all stages of the life cycle in the Global assay by comparing *MGG_Crh2* expression in representative tissues. These were 8 hpi (immature appressoria), 48 hpi (*in planta* growth), vegetative growth (CM) and perithecia, relative to 0 hpi (dormant spores).

Two technical replicates were used per reaction. When possible, the plate design carried all three biological replicates for target and control sample respectively, on the same plate; and, for each sample analysed, *MGG_Crh2* and two validated reference genes were deposited onto the same plate. For expression analysis involving more than one 96-well plate, comparison of samples across different plates was achieved by incorporating inter-run calibration (IRC) wells for each gene in equivalent wells within each 96-well plate. The generated Ct values for samples from multiple runs were imported into MxPro QPCR Software or ‘qBase-Plus Basic Version 2.5’ for IRC calibration [51].

The calibrated values from multiple runs and raw Ct values from one well, were exported into a Microsoft Excel file and analysed separately according to the recommendations by Willems et al. (56). In particular, following their protocol we log transformed, mean centered, and autoscaled the data from three biological replicates, before evaluating the significance of up/down regulation at the 0.05 level by observing whether or not the 95% confidence interval for fold-change increase/decrease in expression contained a fold-change value of 1 (no change). Significant up/down regulated results are indicated by an asterisk on all graphs.

## Results & Discussion

Nine candidate reference genes (Table 1) were selected on the basis of being (a) previously used in *M*. *oryzae* gene expression studies [42, 46, 47], (b) from several essential functional groups (protein translation, glycolysis pathway, cytoskeleton) and (c) expression data in the publicly available high throughput-superSAGE transcript profiling COGEME database of *M*. *oryzae* [49]. A base mean ratio above 10 in the COGEME database was interpreted as indicating that gene expression was unstable, and the gene was unsuitable to be a candidate reference gene. Genes with a base mean ratio below 10 were selected for subsequent sensitive analysis (qRT-PCR using GeNorm calculations) to identify the most stable gene.

### Primer Specificity and Efficiency Check

Each primer specificity assay yielded a single amplicon of the expected size for the primer sets tested (Table 1 and S1 Fig–S2 Fig). There was no amplification, or high Ct values (>35 Ct), for both non-template controls, minus reverse transcriptase (-RT) and the host-only control (BL). This showed that the reagents were free from contamination as there was no amplification of gDNA and no non-specific amplification of plant cDNA. Calculation of primer efficiencies using five-fold dilution of pooled complementary DNA (cDNA) for all nine HKG primers and *MGG_Crh2* gave r^2^>0.99 and 80–110% efficiency (E) values (S3 Fig).

### RNA and cDNA Quality and Quantity Check

The RNA Integrity Number (RIN) values derived from pure fungal RNA samples (dormant spores, perithecia, and vegetatively-grown tissues) were >7.5 (S4 Fig), whilst those containing a mix of pathogen and host plant tissues had RIN values >6.0 (S5 Fig). The lowered RIN values from plant-derived RNA were attributable to the presence of both pathogen and host leaf RNA (contributing cytosolic and chloroplastic ribosomal RNA), giving rise to additional multiple peaks during electrophoretic separation. Consequently, the slightly lowered RIN number is not due to actual nucleic acid degradation (RIN <6.0), but due to the presence of additional peaks, which increases the calculated total area below the electrophoretic graph, a measurement required for the RIN algorithm [56, 57].

### Expression Stability of Candidate Reference Genes

**Fig 1:**
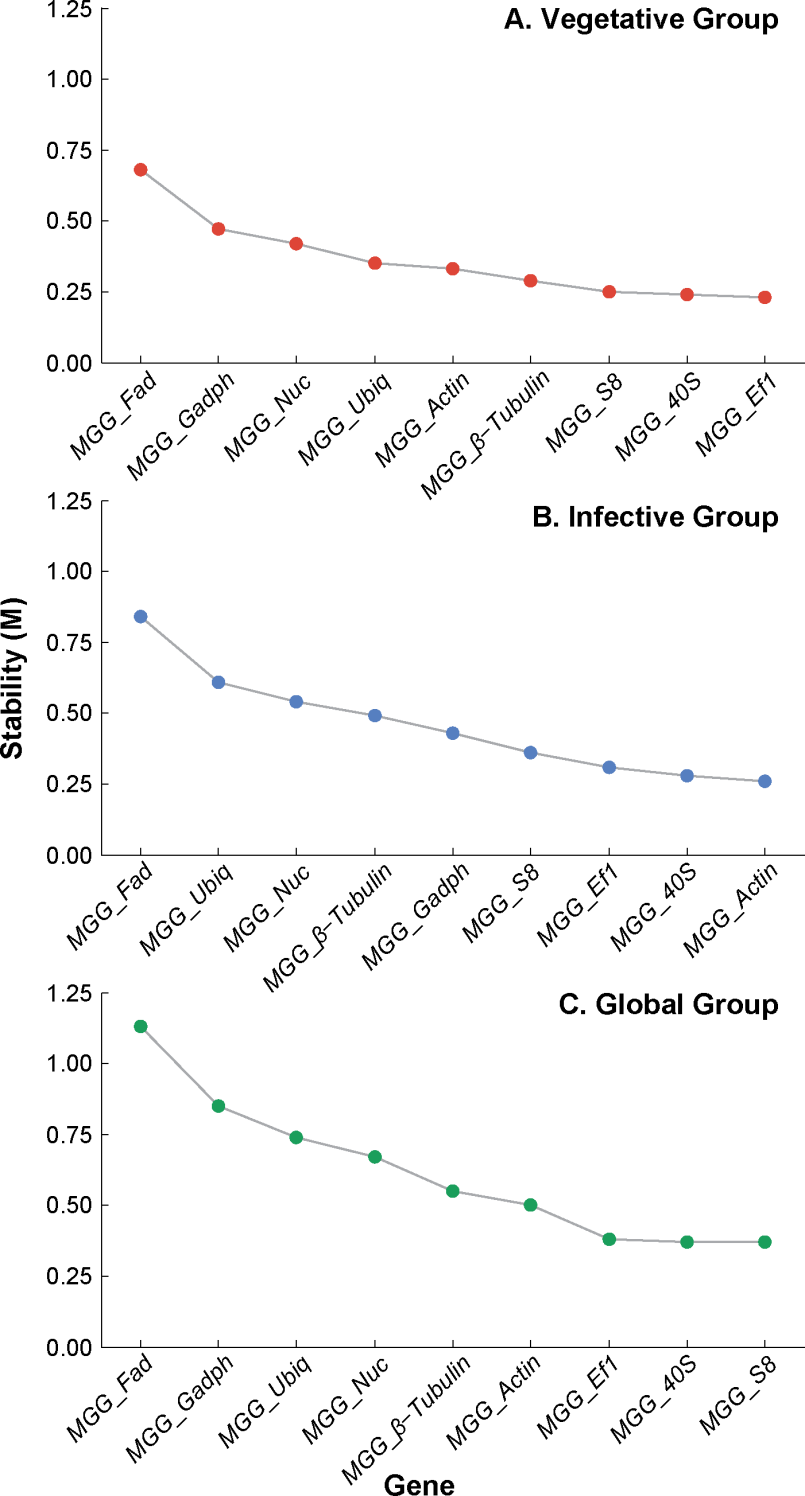
Ranking of nine candidate reference genes in *M*. *oryzae* according to the calculated average expression stability (M). Sequential removal of the least stable reference gene improves average expression stability, indicated by lower values of M. The cut-off for an unstable gene was taken to be M ≥ 1. The analysis was repeated for three groups of tissues: A) Gene stability (M) values for genes in the Vegetative group; B) Gene stability (M) values for genes in the Infective group and; C) Gene stability (M) values for genes in the Global group.

**Fig 2:**
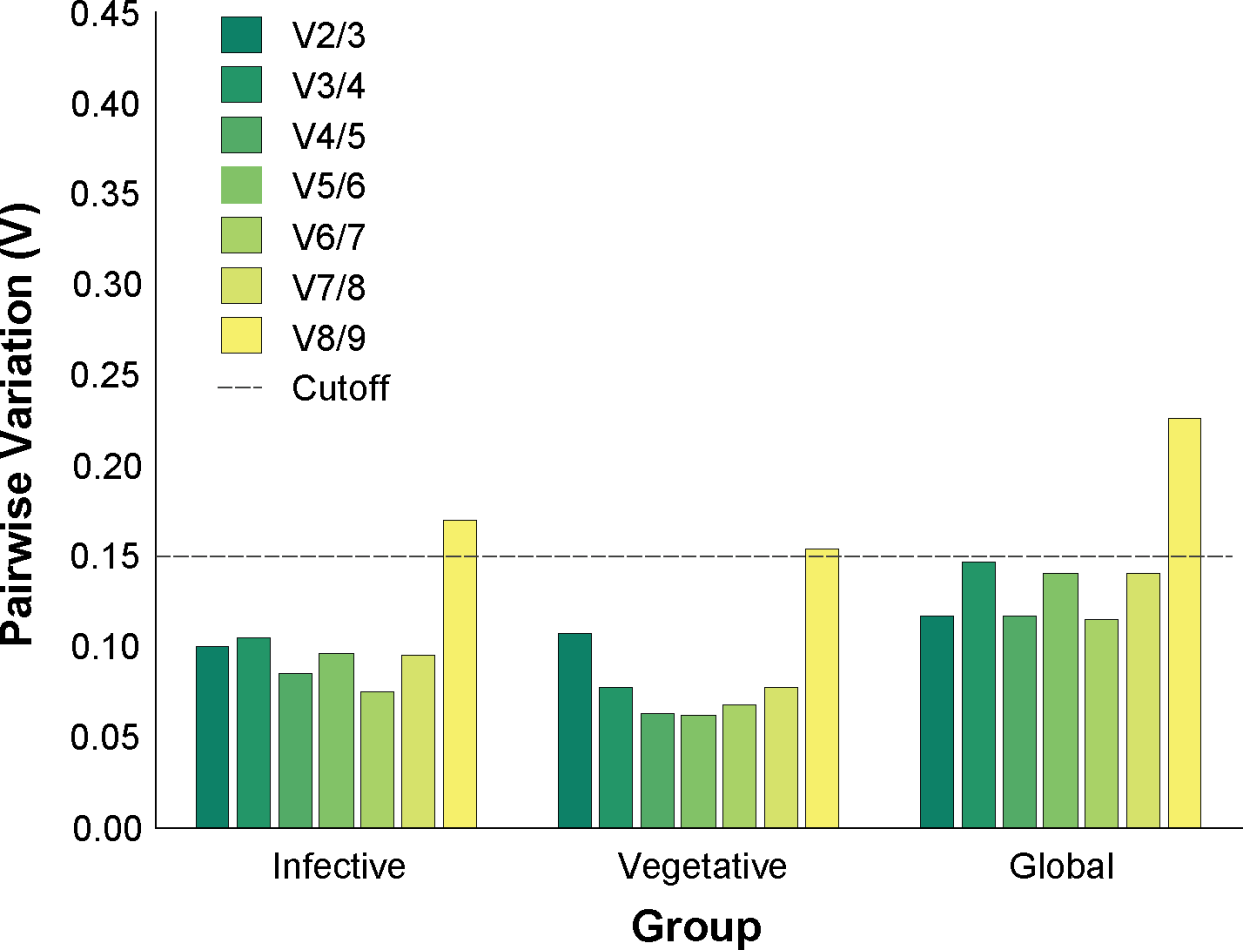
Determination of the optimal number of reference genes for accurate normalisation. Graphs show calculated pairwise variation, *V*_*n/(n+1)*_, between normalisation factor *NF*_*n*_ and *NF*_*n+1*_ for a total of nine candidate reference genes. The optimal number of reference genes for accurate normalisation was taken to be the smallest number for which *V* was less than a cut-off of 0.15 [53]. The analysis was repeated for the three designated groups: A) Infective; B) Vegetative, and; C) Global.

The gene stability (M) values of the nine candidate reference genes, assayed separately for each group of tissues (Vegetative, Infective, and Global), are shown in Fig 1. Candidate genes with the lowest M value were the most stably expressed, whilst genes with the highest M value were least stable. The minimum number of reference genes required for reliable and accurate normalisation for each tissue group was determined as two genes, with a “cut-off” value was set at 0.15 [53] as shown in Fig 2. The gene stability (M) and pairwise variation (V) measurement data were used to identify the two best-performing reference genes for each group; *MGG_Ef1* and *MGG_40s* for the Vegetative Group; *MGG_40s* and *MGG_Actin* for the Infection Group, and *MGG_Ef1* with *MGG_S8* or *MGG_40s* (same M value) for the Global Group.

### Reference Gene Validation

Our recommendations for use of the named reference genes for the given tissues are robust but we advocate caution in extrapolation to untested experimental conditions, such as in the use of different exogenous stresses (*eg*. temperature, pH or heavy metal stress). Moreover, inferred co-regulation is an important consideration in the choice of reference genes and should be avoided, if possible. Here, we selected at least two candidate genes from key functional classes, that is, the glycolysis pathway (*MGG_Fad* & *MGG_Gapdh*), protein synthesis (*MGG_Ef1*, *MGG_S8* & *MGG_40s*), cytoskeleton assembly (*MGG_β-Tubulin* & *MGG_Actin)* and protein degradation (*MGG_Ubiq)*. For the Infective Group, the two most stable genes, *MGG_40s*, and *MGG_Actin* belong to two distinct functional groups. However, for the Vegetative and Global Groups, genes belonging to the protein synthesis pathway proved most robust in stability, being unaffected by changes to environmental stressors or fungal growth phases. In this case, it is more important to use a set of validated stable genes instead of prioritising genes from different biological pathways. Furthermore, for the Vegetative Group, the two most stable genes belong to protein synthesis pathways with exclusive functions: *MGG_Ef1* and *MGG_40s*, therefore have little risk of co-regulation [58, 59]. In the Global Group, the most stable genes lie in the protein synthesis pathway. Here, as *MGG_40s and MGG_S8* both form one 40S functional subunit, we recommend instead *MGG_Ef1* (M = 0.38), used in conjunction with *MGG_40s* (M = 0.37) or *MGG_S8* (M = 0.37). Here, the M value differences are very small, suggesting that all three genes are similarly stable and thus this will not significantly affect normalisation reliability.

Thus far, a single study has attempted identification and validation of *M*. *oryzae* reference genes for qRT-PCR [27]. In that study, Park et al. (2013) chose seven candidate genes, three of which are used herein and can therefore be compared directly, namely *MGG_β-Tubulin*, *MGG_Actin* and *MGG_Gapdh*. Based on GeNorm analysis, Park et al. (2013) ranked their seven genes across tissue sample types that concur with our Global Group, whereby *MGG_Gapdh* has lower stability (high M value), with *MGG_Actin* and *MGG_β-Tubulin* being more stably expressed (low M value). Our combined findings also correspond for the Infective Group, whereby *MGG_Actin* is more stable than *MGG_Gapdh* and *MGG_β*-tubulin. This consistency between studies confirms the reproducibility and reliability of qRT-PCR when quality control practices were adhered to. In addition to the three genes shared by both studies, here we introduce a validated set of more robust genes, particularly those belonging to the protein synthesis pathway (*MGG_Ef1*, *MGG_40s*, and *MGG_S8*) that can be used for gene expression studies in *M*. *oryzae*. This study also demonstrates that for accurate and reliable normalisation of *M*. *oryzae* genes across all tissue groups (Infective, Vegetative, and Global), the optimal number of reference genes to use is two and not one as used in many *M*. *oryzae* gene expression analysis studies hitherto.

### Gene of Interest (GOI): *MGG_Crh2* Expression Profile

**Fig 3:**
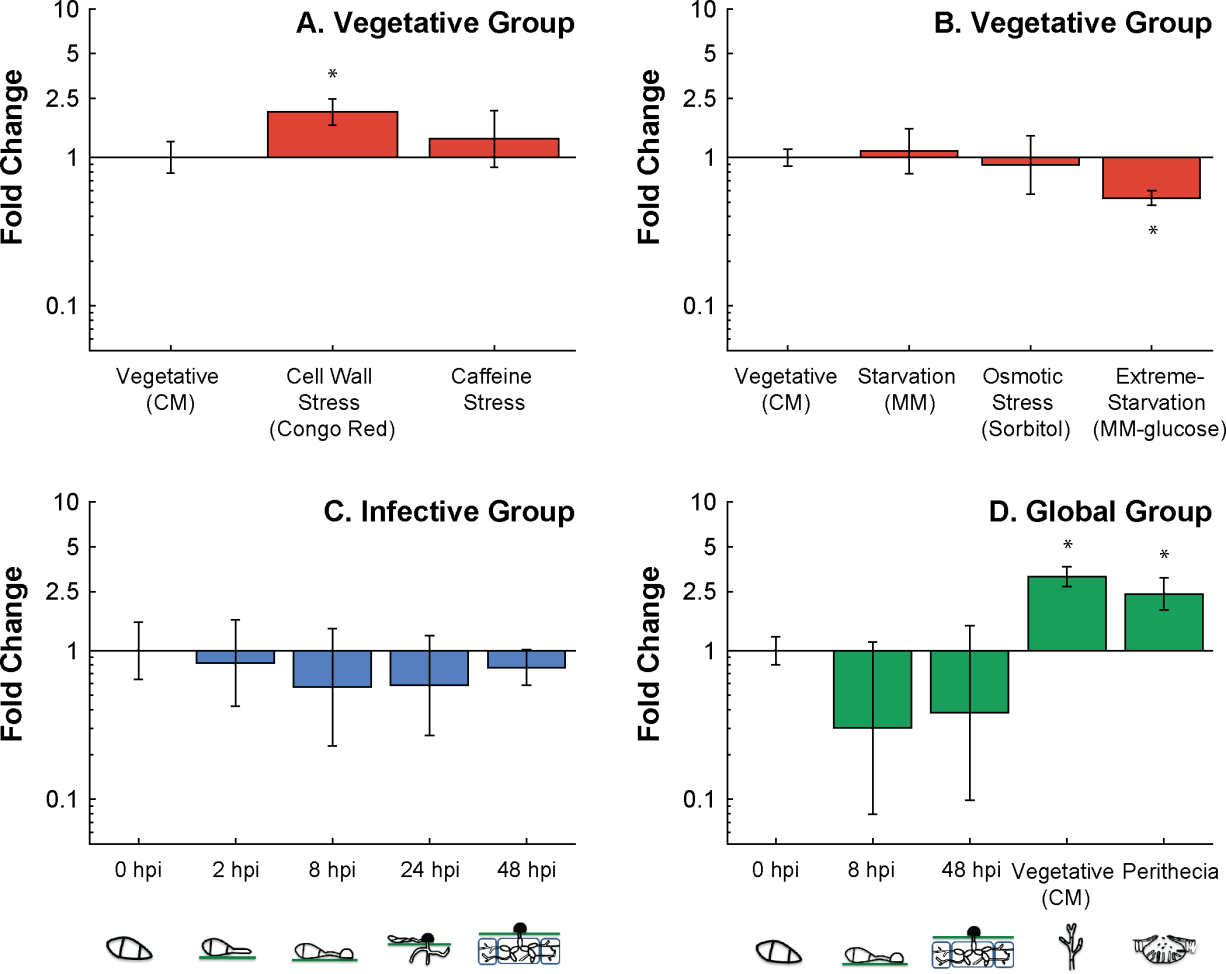
The expression profile of *M*. *oryzae MGG_Crh2* cell wall remodelling gene measured by qRT-PCR. A total of 12 different tissue samples were assigned into three distinct tissue groups: Vegetative, Infective, and Global group each containing 5–6 tissue types. Each graph was assayed and analysed separately. Each bar corresponds to three biological replicates of the same tissue. Error bars are at 95% confidence interval. Panel A. Vegetative group. The expression of *MGG_Crh2* when vegetatively grown under cell wall stress induced by Congo Red (100 μg/mL) and caffeine (25 mM) relative to growth under optimal conditions in CM. Reference genes *MGG*_*Ef1* and *MGG_40s* were used as the normalising factor. Panel B. Vegetative group. The expression of *MGG_Crh2* when vegetatively grown under starvation (MM), osmotic stress (Sorbitol 1M) and extreme starvation (MM-glucose), relative to growth under optimal conditions (CM). Reference genes *MGG*_*Ef1* and *MGG_40s* were used as the normalising factor Panel C. Infective Group. The expression of *MGG_Crh2* at 2 hpi (germ tube), 8 hpi (immature appressorium), 24 hpi (penetration and early invasive growth), and 48 hpi (*in-planta*) relative to 0 hpi (dormant spores). Reference genes *MGG*_*Actin* and *MGG_40s* were used as the normalising factor Panel D. Global group. The expression of *MGG_Crh2* at 8 hpi (immature appressorium), 48 hpi (*in-planta*), vegetative mycelium (CM) and in perithecia, relative to dormant spores (0 hpi). Reference genes *MGG*_*Ef1* and *MGG_40s* were used as the normalising factor.

Reciprocal blast search using the *S*. *cerevisiae* gene *Crh1* protein sequence revealed *MGG_00592* as the best-hit gene and most probable orthologue, with a cut-off value of e^-10^. It is hereby named *MGG_Crh2*. We exploited the stable pairs of recommended reference genes as normalising factors to trace the expression profile of the cell wall remodelling gene *MGG_Crh2*, an orthologue of *S*. *cerevisiae* Crh1 gene (S1 Dataset). In the vegetative assay, we used *MGG_40s* and *MGG_Ef1* as reference genes and observed that *MGG_Crh2* expression responded to cell wall stress imposed by Congo Red, being elevated some 2-fold (p<0.05), and was downregulated under extreme starvation (MM-glucose) (Fig 3, Graph A and B) but was not affected by growth under cell wall stress imposed by caffeine, hyperosmotic stress (Sorbitol), or by moderate starvation (MM).

In *S*. *cerevisiae*, *Crh1* has previously been reported to be upregulated 2–4 fold in the presence of Congo Red [26], and cell wall stress caused by Congo Red was shown to activate the cell wall integrity pathway directly via the phosphorylation of Rlm1 [26]. Additionally, the lack of response to cell wall stress induced by caffeine is an interesting observation. Caffeine is a compound known to activate the cell wall integrity pathway leading to phosphorylation of the *S*. *cerevisiae* Mpk1 and shown to alter yeast cell wall architecture upon transient exposure to caffeine [60]. Further studies need to be conducted to investigate whether MoMig1p [61], an orthologue of *S*. *cerevisiae* Rlm1p, indeed regulates the expression of *MGG_Crh2* under cell wall stress and if so, whether it is selective to certain types of stressors.

In the infective assay, *MGG_40s* and *MGG_Actin* were used as the normalising factor with expressions measured relative to 0 hpi (dormant spores). Results in Fig 3 (Graph C) showed that *MGG_Crh2* is not regulated during germling development as its expression in each tissue type (germ tube, immature appressorium, penetration and early invasive growth as well as *in planta* growth) was not significantly different from dormant spores. This is an interesting outcome, given that in other *M*. *oryzae* cell wall protein studies cell wall biosynthesis and remodelling enzymes such as the chitin synthase *Chs7* gene [62] and two *Gel* orthologues (MGG_11861 and MGG_08370) [63] were found to be upregulated during appressorium development and for the former, mutants were non-pathogenic. Interestingly, studies of the human fungal pathogens *Candida albicans* and *Aspergillus fumigatus* showed that the orthologues of *S*. *cerevisiae* Crh1p (Crh11p and Crf1p respectively) are specifically detected by the mammalian host immune response system [64–67]. It is therefore conceivable that the lack of upregulation of *MGG_Crh2* in infective structures aids *M*. *oryzae* in evading detection. The literature is currently devoid of information on plant immune responses elicited by recognition of secreted fungal cell wall remodelling enzymes. There is however, evidence showing that the cell wall of *M*. *oryzae* is remodelled during infection to ‘mask’ the fungi with α-1,3 glucan [68], suggesting that the fungi is indeed under selection to evade detection. This unexplored area of plant-pathogen interactions is worth exploring in future work.

The theory is further supported by results in the Global assay (Fig 3 graph D). In this assay, *MGG_Crh2* gene expression in tissues representative of each stage of the life cycle was each measured relative to dormant spores (0 hpi). Data analysis showed that this gene is most highly expressed during vegetative growth by up to 3-fold higher (p<0.05), followed by 2.4-fold higher in perithecia and lowest expression during the infective stages, as shown in Fig 3, during appressorium development (8 hpi) and *in planta* growth (48 hpi).

In summary, this expression study has shed light into the regulatory profile of the transglycosylase cell wall remodelling gene, *MGG_Crh2*, which was found to be most highly expressed during vegetative growth with down-regulation during the development of infective structures and *in planta* growth. We also have identified an optimum set of control genes for studying gene expression across the life-cycle of *M*. *oryzae* using qRT-PCR. Further work is required to expand this gene-set to cover a broader range of growth conditions, such as varied exogenous stresses, but this robust approach sets a standard of practice for qRT-PCR gene analysis which can only increase our understanding of this devastating rice pathogen.

## Supporting Information

S1 Dataset Data analysis for the expression profile of *MGG_Crh2*. (XLSX)

S1 Fig Primer specificity analysis by PCR and subsequent size separation by gel electrophoresis for each targeted gene. Products were PCR amplified using pooled cDNA template from all samples and size separated alongside GeneRuler Ultra Low Range DNA Ladder (10–300 bp). Gel electrophoresis was conducted in 2% agarose (SIGMA-Aldrich).
(TIF)

S2 Fig Primer specificity analysis by melt-curve graphs for each targeted gene. Graphs show PCR product dissociation curves for ten primer pairs used in this analysis using pooled cDNA of WT from all samples used. The melt curve data was obtained from the denaturation of amplified PCR product executed at the end of a qRT-PCR run, by temperature increment.
(TIF)

S3 Fig Real-Time PCR primer efficiency analysis. The cycle threshold (ct) of ten primer pairs for candidate reference genes plotted against a five-fold dilution of pooled cDNA from all samples analysed. Each qRT-PCR reaction had two technical replicates, therefore the cycle threshold (ct) value above is an averaged data. All reactions conducted on the same 96-well plate. The slopes and r^2^ values were calculated using a regression line across four-points.
(TIF)

S4 Fig Sample RNA quality analysis by electrophoretic separation for samples containing only pathogen RNA. Each electrophoregram comprise of RNA of fungal tissue grown vegetatively under various conditions, dormant spore (0 hpi) or perithecia. Each graph showed the presence of two sharp peaks, corresponding to two bands on the right side of each graph, indicating good quality RNA.
(TIF)

S5 Fig Sample RNA quality analysis by electrophoretic separation for samples containing RNA from both host and pathogen. Each electrophoregram comprise of RNA taken from host leaf tissue inoculated with fungal spores (in 0.2% gelatine) at various hours post inoculation (hpi). Control leaf comprise of plant leaf sprayed with 0.2% gelatine. Each graph above showed the presence of two sharp peaks, corresponding to two bands on the right side of each graph, indicating undegraded RNA. Additional peaks correspond to chloroplastic ribosomes abundant in samples derived from host leaf tissues.
(TIF)
