# Supplementary figures and images for: Validation of Reference Genes for Robust qRT-PCR Gene Expression Analysis in the Rice Blast Fungus Magnaporthe oryzae
Source: PLoS One. 2016 Aug 25;11(8):e0160637. doi: 10.1371/journal.pone.0160637 (PMC4999194; doi:10.1371/journal.pone.0160637)

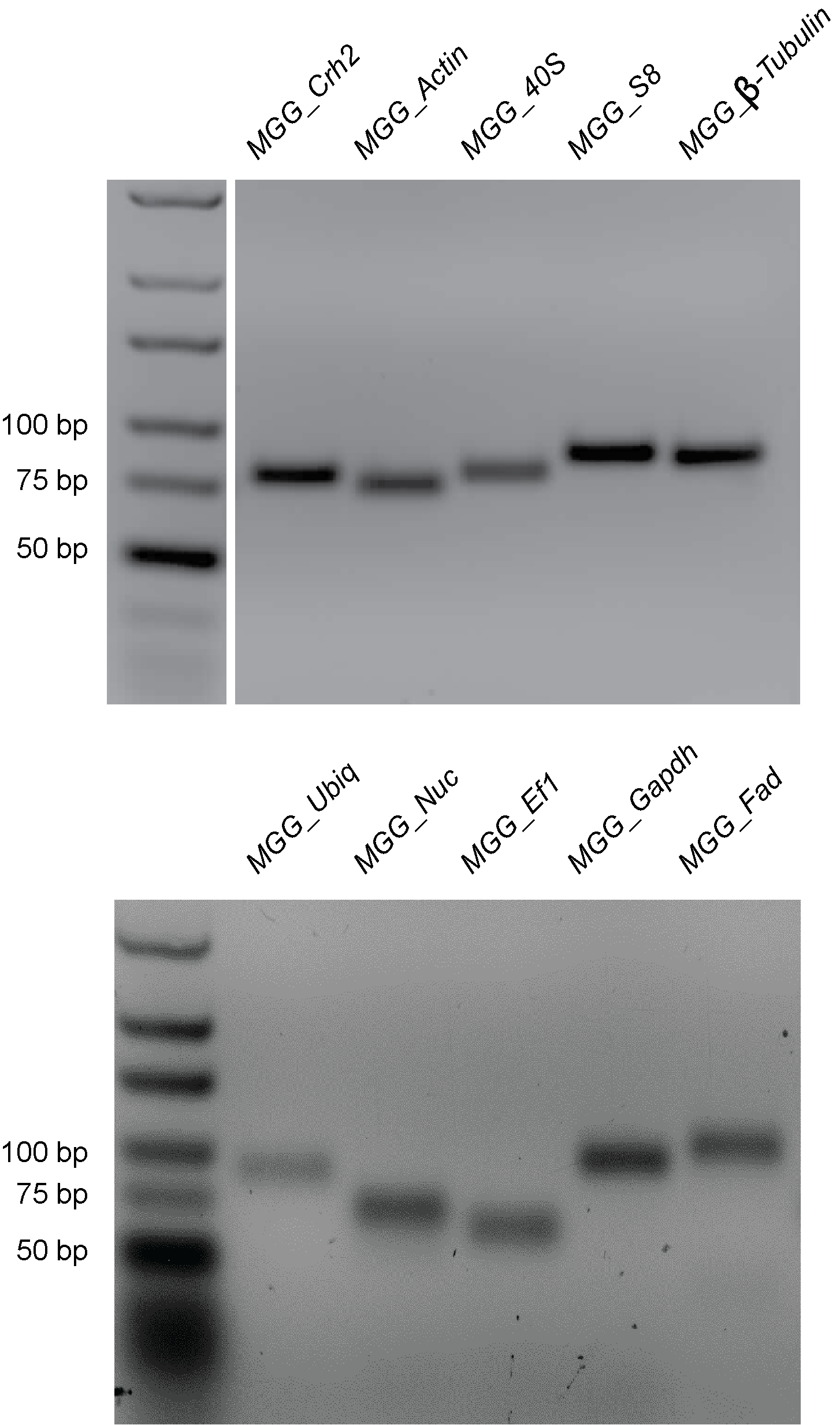

Supplement: S1 Fig — Products were PCR amplified using pooled cDNA template from all samples and size separated alongside GeneRuler Ultra Low Range DNA Ladder (10–300 bp). Gel electrophoresis was conducted in 2% agarose (SIGMA-Aldrich). (TIF) [file pone.0160637.s002.tif]

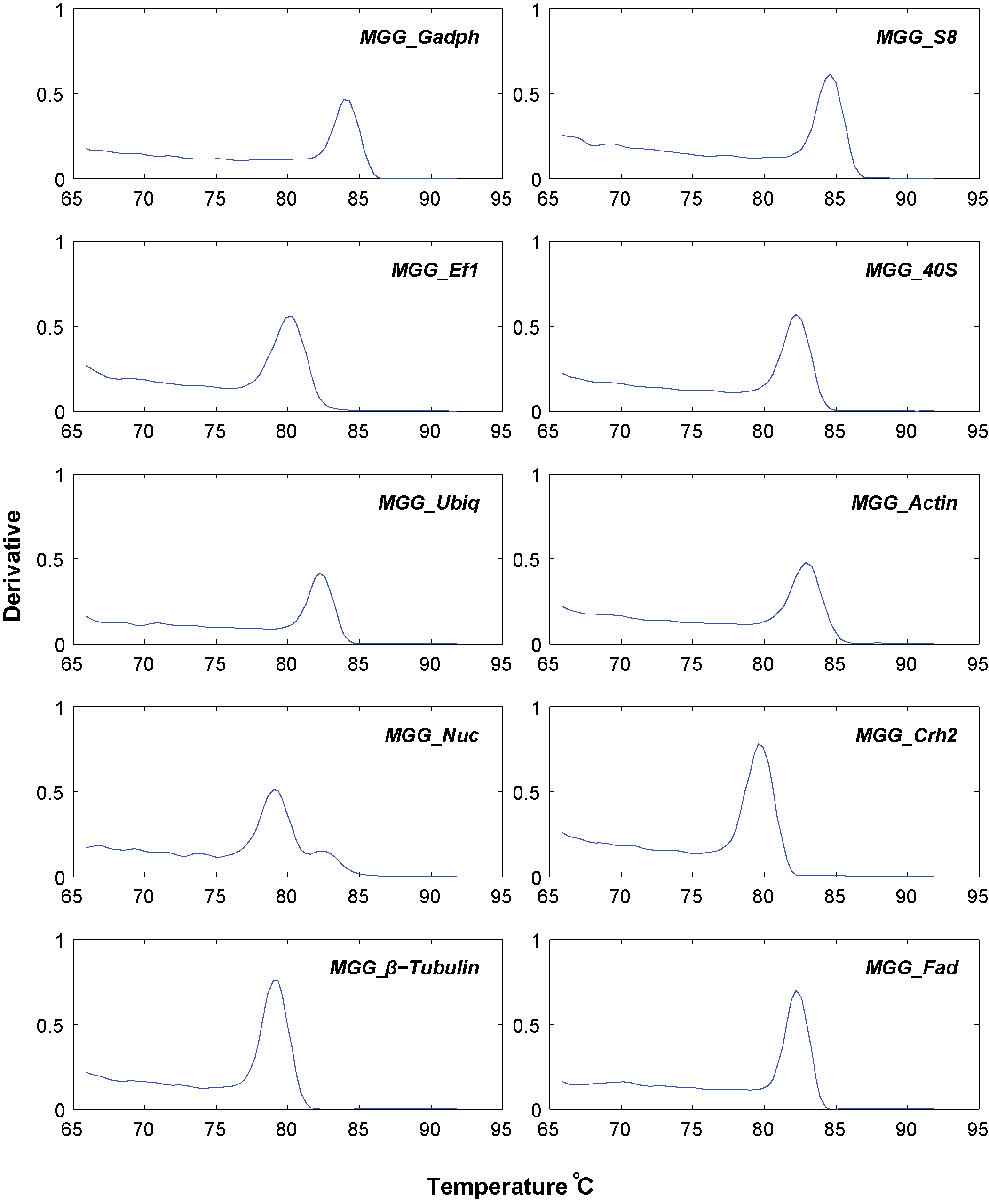

Supplement: S2 Fig — Graphs show PCR product dissociation curves for ten primer pairs used in this analysis using pooled cDNA of WT from all samples used. The melt curve data was obtained from the denaturation of amplified PCR product executed at the end of a qRT-PCR run, by temperature increment. (TIF) [file pone.0160637.s003.tif]

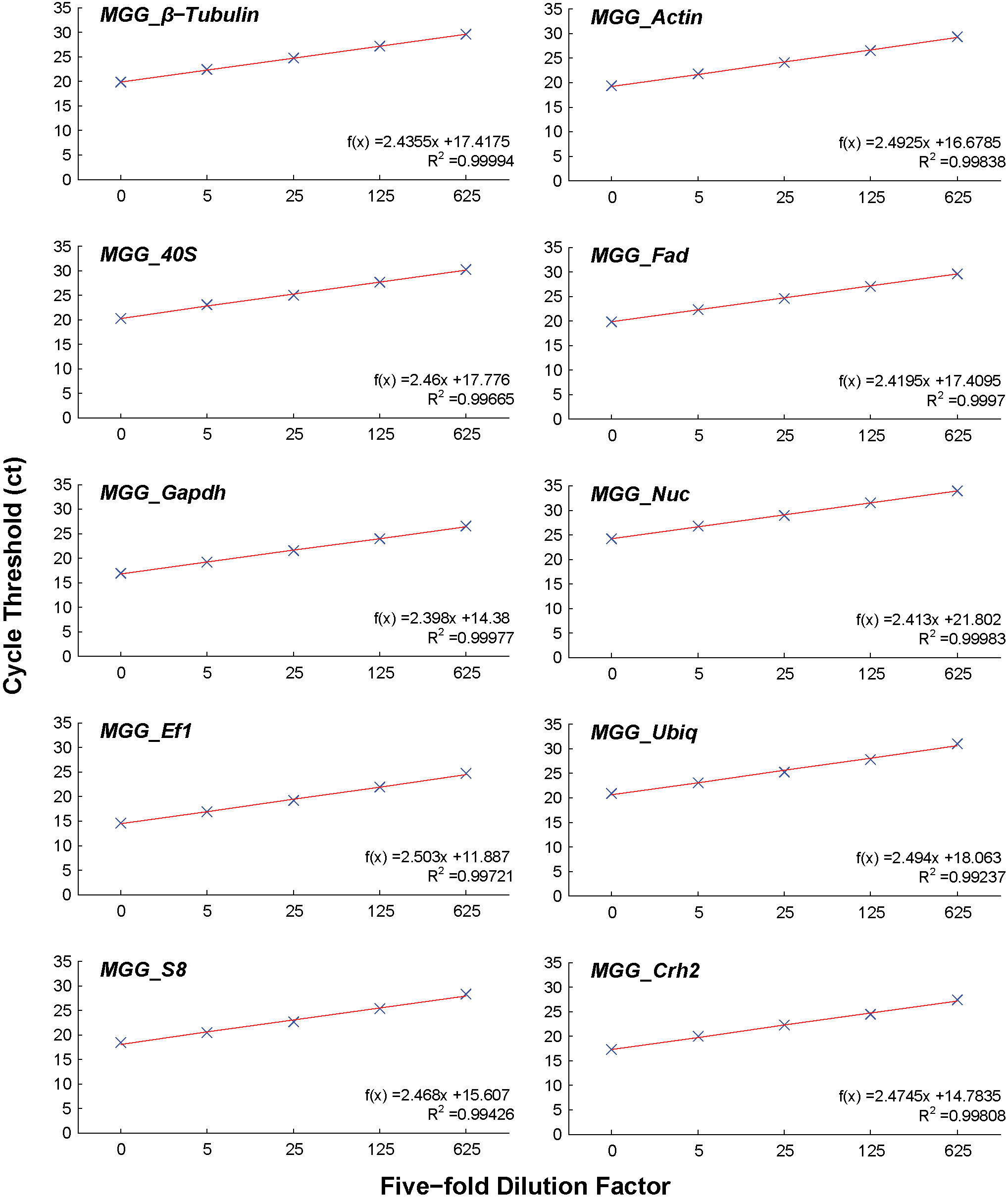

Supplement: S3 Fig — The cycle threshold (ct) of ten primer pairs for candidate reference genes plotted against a five-fold dilution of pooled cDNA from all samples analysed. Each qRT-PCR reaction had two technical replicates, therefore the cycle threshold (ct) value above is an averaged data. All reactions conducted on the same 96-well plate. The slopes and r2 values were calculated using a regression line across four-points. (TIF) [file pone.0160637.s004.tif]

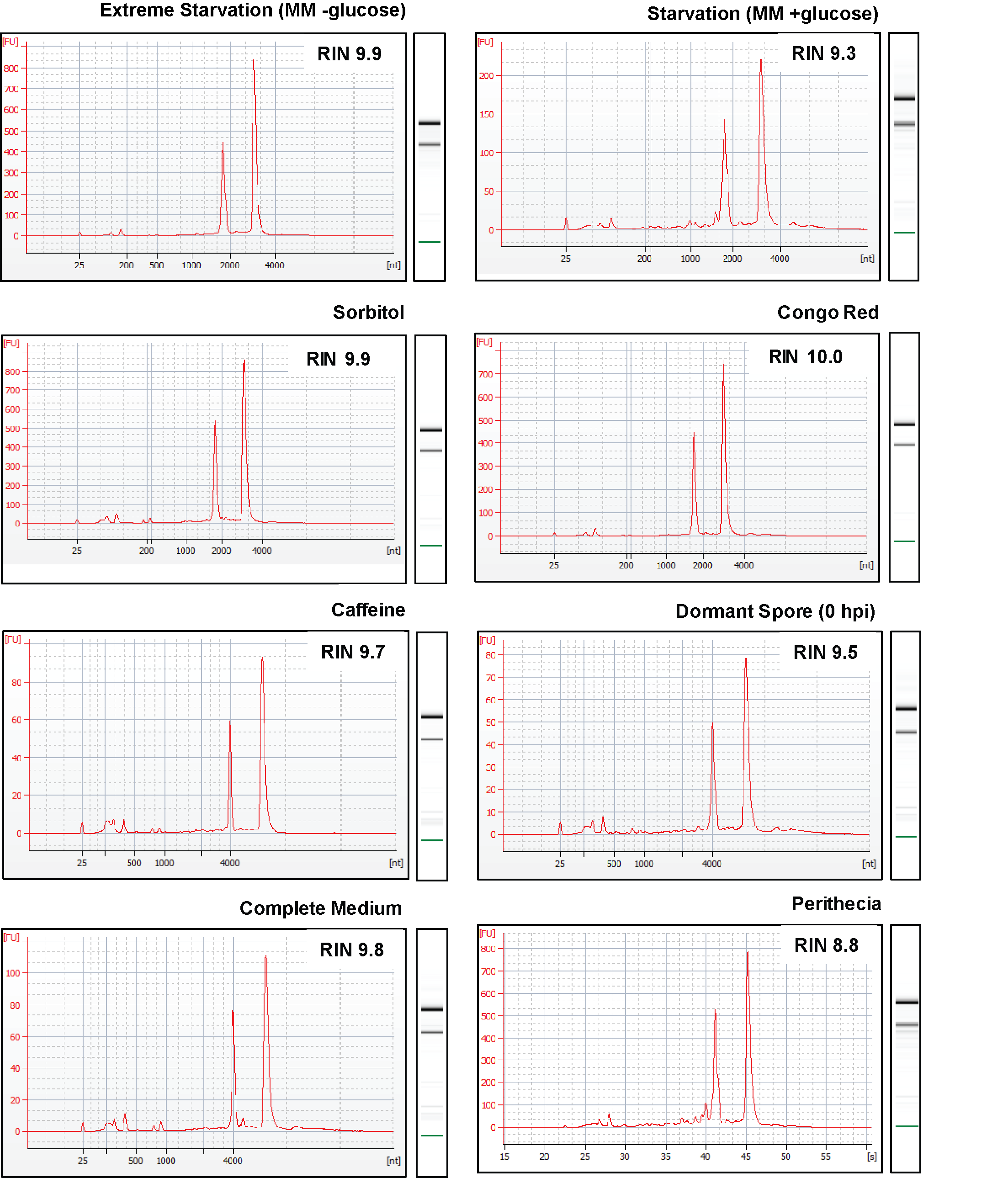

Supplement: S4 Fig — Each electrophoregram comprise of RNA of fungal tissue grown vegetatively under various conditions, dormant spore (0 hpi) or perithecia. Each graph showed the presence of two sharp peaks, corresponding to two bands on the right side of each graph, indicating good quality RNA. (TIF) [file pone.0160637.s005.tif]

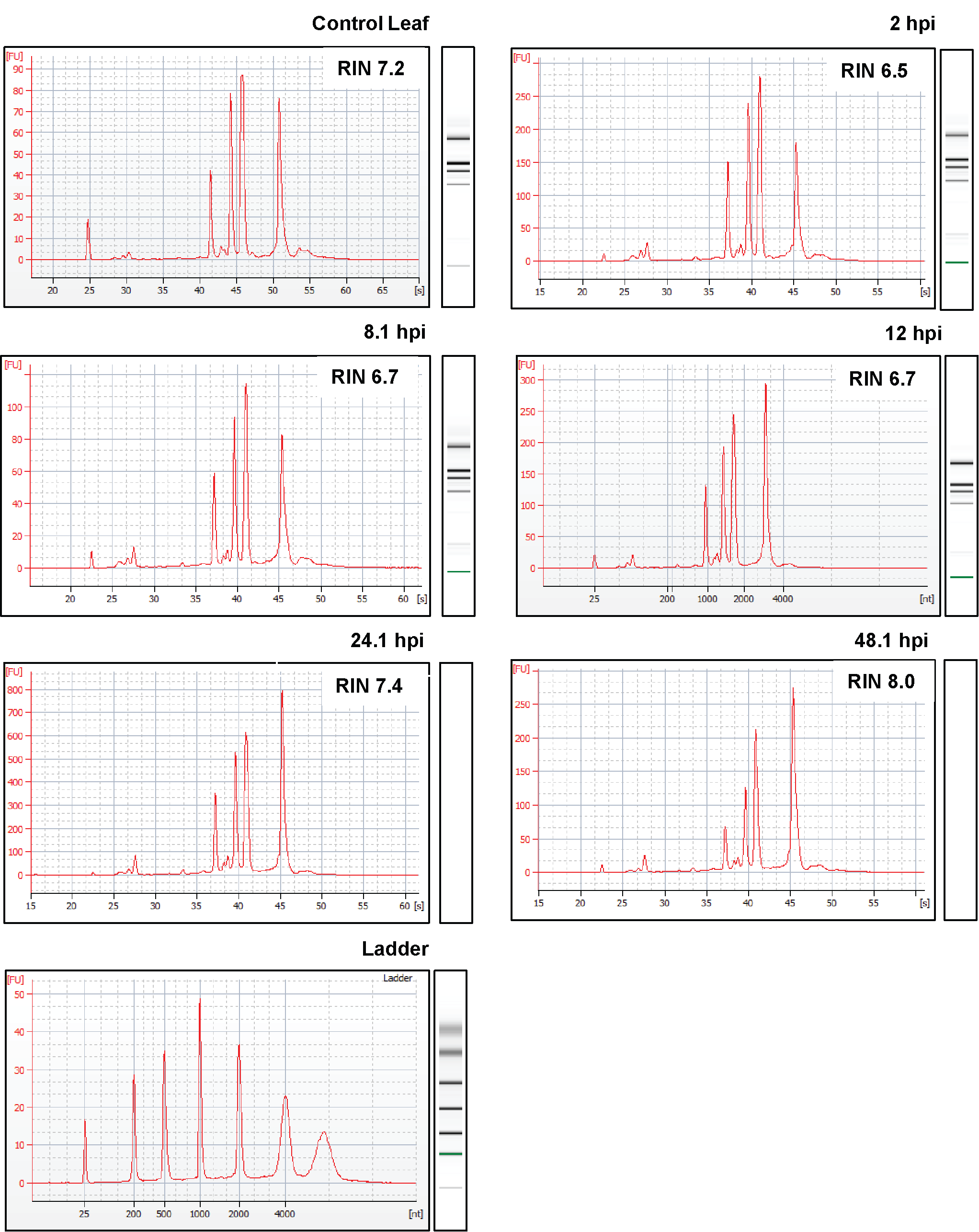

Supplement: S5 Fig — Each electrophoregram comprise of RNA taken from host leaf tissue inoculated with fungal spores (in 0.2% gelatine) at various hours post inoculation (hpi). Control leaf comprise of plant leaf sprayed with 0.2% gelatine. Each graph above showed the presence of two sharp peaks, corresponding to two bands on the right side of each graph, indicating undegraded RNA. Additional peaks correspond to chloroplastic ribosomes abundant in samples derived from host leaf tissues. (TIF) [file pone.0160637.s006.tif]
